# Stop play: key takeaways on physical safety awareness in healthcare simulation education

**DOI:** 10.1186/s41077-026-00421-2

**Published:** 2026-03-17

**Authors:** Tatiana Lubin, John Cruz, Komal Bajaj, Michael Meguerdichian, Katie Walker

**Affiliations:** 1 Institute for Medical Simulation and Advanced Learning, CUNY School of Medicine, Bronx, NY USA; 2 Albert Einstein College of Medicine, Bronx, NY USA; 3 Institute for Medical Simulation and Advanced Learning, Bronx, NY USA; 4 Columbia Vagelos College of Physicians and Surgeons, New York City Health + Hospitals: Harlem Hospital Center, New York, NY USA; 5 Mater Hospitals and Healthcare, Brisbane, Queensland Australia

**Keywords:** Simulation Physical Safety, Situational Awareness, Safety Phrase

## Abstract

Healthcare simulation is a practice used to train healthcare workers and refine their clinical and behavioral skills. This practice is underpinned by its safe learning environments, where participants can learn from their mistakes without fear of patient harm. A safe learning environment in simulation education encompasses physical and psychological safety. To date, physical safety has rarely been discussed in the literature. This criterion aims to act as a catalyst to motivate situationists to think explicitly about physical safety when creating and conducting simulation experiences. Drawing from both experience and evidence base, this paper looks to build safety awareness, and guidance for developing and implementing explicit physical simulation safety protocols & best practices, as well as outline physical simulation safety training to prevent adverse events during simulation activities. We believe that prevention is better than a cure.

## Introduction

A large metropolitan-based simulation center annually conducts a simulation-based teamwork and communication course for incoming internal medicine and surgery residents. Verbal de-escalation is a primary learning objective of the course and is highlighted in a scenario focused on interactions with an agitated patient. The scenario escalates when a patient actor, acting aggressive, charges toward an embedded participant nurse. Even though the nurse actor had played this role many times, in this instance, she became nervous, lost her footing, and fell backward to the floor. She felt pain shooting through her hand, and one of her colleagues bent down to ask if she was ok. She indicated she wasn’t. Confusion ensued, and the simulation was halted. There was no universally known process to deal with a physical injury in this medical simulation environment. After multiple phone calls, the staff worked together to get the injured actor to the Emergency Department. X-rays confirmed a real injury had occurred, a broken radius. Until today, true physical injury was not considered a part of educational experiences.

Safety in learning environments is something that is assumed, and typically an afterthought that is in need of re-evaluation. The National Center on Safe Supportive Learning Environments proposes a vision, specific to physical safety, where students should attend and fully engage in learning experiences that are without distraction related to concerns about physical safety [1]. As we consider safety within healthcare simulation, much attention has been directed to psychological safety, through establishing and maintaining a safe container [2, 3], but few have addressed physical safety. As healthcare simulation programs recreate hospital settings, simulated events can pose similar injury risks associated with working in hospital environments [4]. Considering the risk of hospital-related physical injury, it is crucial to clarify how to safely operate in experiential learning environments with a physical safety focus.

Experiential learning environments span a variety of modalities including manikin and human simulationist-based scenarios, virtual reality, augmented reality, computer-based and others, all with specific safety risk profiles. Knowing that experiential learning is, at times, delivered on clinical units, an even higher awareness of safety risk needs to be factored in when simulation is delivered ‘in-situ,” as lines between simulated and real practice may be blurred [5]. Even though these theoretical risk for injury seem high, physical incidents within simulation environments appear to be rare [6]. It is unclear if the lower incident rate of injury in healthcare simulation relative to hospital care settings is due to under-reporting and a lack of platforms to share adverse physical safety events for organizations [7]. To begin addressing this, the Foundation for Healthcare Simulation Safety has been making a large effort to build awareness and provide case examples of real safety incidents [8].

The lack of a structured approach to evaluation, reporting and refinement of physical safety poses a significant gap in healthcare simulation. This paper looks to explicitly discuss an adapted conceptual framework, the Donabedian Model, to develop safety approaches that will aid in building awareness, promote understanding and offer physical safety systems and strategies to embed within simulation programs [9]. Lastly, we describe strategies used by our institution to foster safety and describe trainings in support of a culture of safety for simulation programs.

### Adapting the Donobedian model to simulation physical safety

As we consider simulation safety, we can draw on quality improvement tools to guide the development of a structured process for enhancing safety within the Simulation Center. One useful tool is the Donabedian Framework, a well-established model for evaluating healthcare quality [9]. This framework defines quality through three key components: structure, process, and outcomes [10]. Depending on the quality focus, structure refers to the organization’s physical environment, human resources and systems supporting that focus. Process describes the action and workflow upholding quality. Lastly, outcomes represent the results that emerge from the effectiveness of both process and structure. This model has been applied across varied disciplines ranging from outpatient medicine clinics [10], to Undergraduate Nursing Education quality [11] to quality related to care delivery at pharmacies [12]. Given its widespread use across multiple disciplines to evaluate and improve program quality, it has proven an effective tool that can now be adapted to healthcare simulation.

In applying the Donabedian framework to Healthcare Simulation, structural measures include the physical spaces where simulation takes place, staff qualifications and the equipment used during training. These elements create a foundation that supports an effective simulation learning environment. Process measures address how the processes work to deliver a service, in this context, physical safety, during simulation education. Outcome measures refer to the impact of the processes and if they have met their aim [13]. Structural elements impact processes which will ultimately drive outcomes [14]. Exploring each of these elements and their interaction in the context of simulation physical safety will create a blueprint toward safer learning environments.


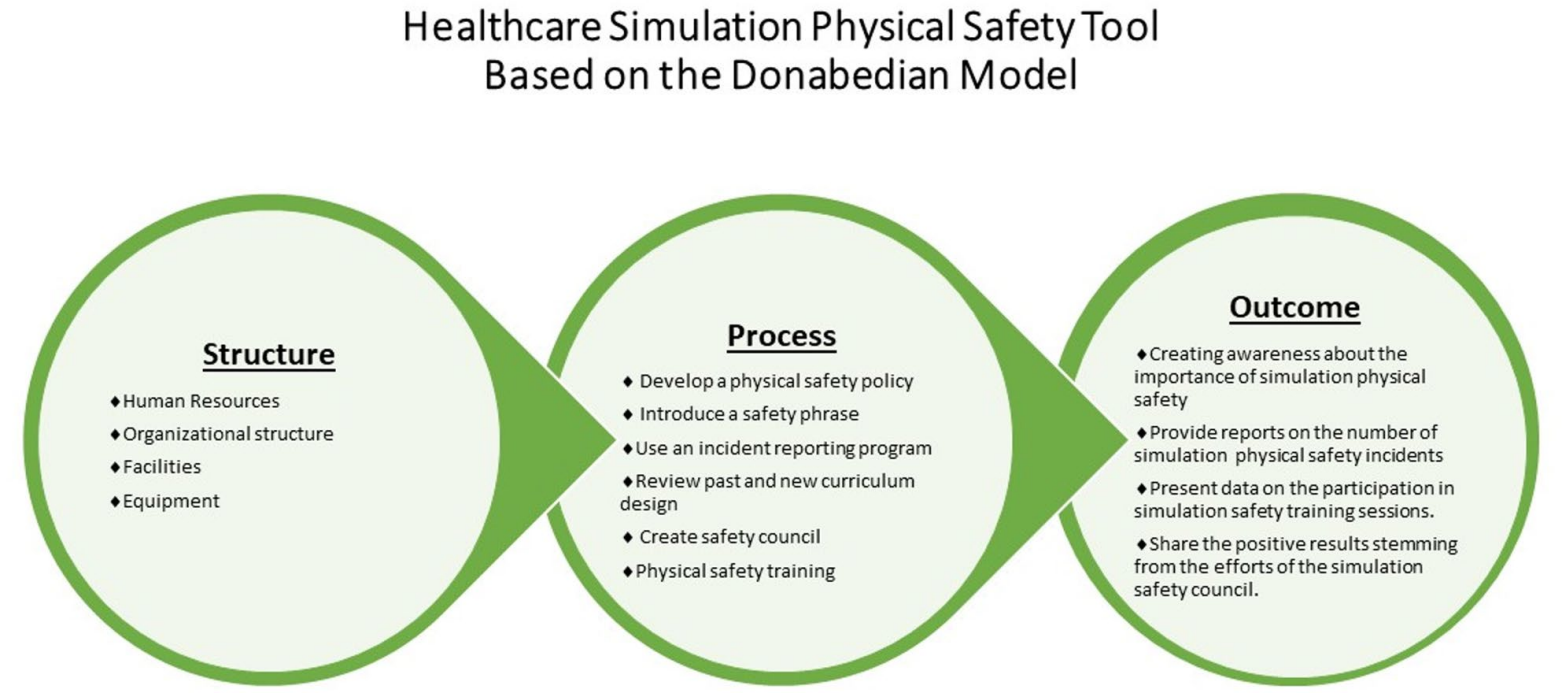


### Structural considerations for enhancing safety in simulation

When considering physical safety, certain structural elements will contribute to the safety risks that exist within the learning experiences. For example, manikins that require tethering, or a power source, leave a cord extending to the wall, at times, create a tripping hazard. Similarly, improper lighting during a suture workshop risk finger sticks as the operators cannot optimally appreciate their needle during handling. Taking an inventory of these structural elements may inform some initial steps that may need to be taken to rising potential risks in reducing hazards. This “Room of Horrors” approach has been gamified for clinical staff honing their skills to identify patient safety risks in environments. Lee et al. [15], in their systematic review concluded that this approach built participant awareness around safety and situational awareness, but further work was needed to understand its long-term effects on safety.

Applying the “Room of Horrors” strategy can be applied to a multitude of structural elements across healthcare simulation. New course work or topics being introduced to an existing space bring new potential risks. Intentionally revisiting pre-existing spaces to consider how the new content can create different hazards can create a plan of action to overcome safety issues. Similarly, bringing a physical safety lens to new space design, can ensure certain risks are not created prior to construction. For example, strategically placing outlets for manikin electrical cords on the floor under a stretcher may avoid the tripping hazard described earlier. Similarly, a visit to an in-situ simulation space can identify potential safety risks prior to engaging in educational interventions. In preparation for a virtual reality experience, a visit to the space prior to the event could illuminate the room is too restrictive or identify equipment the learner might run into while visually impaired by VR goggles.

Staff must be trained to be aware of the safety risks inherent to healthcare simulation education spaces, be encouraged to follow safety protocols, and be empowered to speak up to prevent further safety issues. Through redundancy of practice, staff may become more attuned at identifying risk and as a result develop a more discerning eye when consulted to design new or repurposed clinical space for simulation education.

Equipment and its proper use also contribute to safer environments. Equipment education is imperative, since proper training leads to proper use which avoids accidents, prolongs equipment life, and compliance [16]. With equipment such as defibrillators and ventilators being used in simulation environments, regular servicing by trained specialists ensures that the equipment is properly maintained for safety and reduce the risk of accidental injury. Correct usage and maintenance of the equipment has the added benefit of prolonging equipment life and reducing cost for replacement. Staff and learner compliance is vital to ensure people, products, and processes meet regulatory requirements and ultimately optimizes safe use.

### Process measure and policies for safety

In order to deliver a physically safe educational service, applying processes allows for mobilizing the organizational structure to execute according to the Donabedian Framework. Policies and procedures provide guidelines that facilitate the smooth operations of the facility and help ensure that learners and simulation team members have a shared mental model [17]. Brazil et al. suggest the faculty develop a steering committee and co-create a simulation physical safety policy borrowing from the home institution’s Hospital Safety Management Policy and Procedure. The policy should emphasize the identification of safety and security risks to protect visitors and staff, followed by a systematic approach to eliminate or mitigate the impact of risk [5]. Policy creation can also rely on Reamer’s 10 commandments for Healthcare Simulation Safety, focusing on labeling equipment, building awareness internally and externally, and developing a reporting mechanism and personal experience at the center [6]. The policies serve as a mechanism for planning, standardizing, and documenting the operations aligned with health service standards [5].

Socializing policies moves theory to action. For example, promoting health and safety awareness among hospital healthcare workers decreases the risk of illness and occupational diseases. It creates a more efficient and effective safe hospital environment [18]. Socializing policies within simulation communities can be achieved through leadership communication of policy themes and their importance at meetings, distribution of the policy electronically and ensuring the policy is available in an easily accessed location (i.e. hospital-wide policy page). Our institution engages all employees in policy creation and review to build awareness, create conversation and promote buy-in around all policies, including our safety policy.

Through that communication, it is also important to build awareness around some of the safety behaviors that shape culture. The military enforces the importance of physical safety during their training to ensure everyone knows the safety phrase, “Stopping the Line.” Using this phrase signifies the person holds values aligned with respect, quality, safety, and integrity [19]. Borrowing from their safety culture, our institution applies “Stop Play” as its safety phrase. The phrase halts all activity to inform all participants that a risk-averse situation exists. This concept is introduced at the start of all courses so there is a shared mental model of awareness among faculty and learners.

Another process example taken from our institutional playbook addresses what happens after an injury has occurred. Once a potential safety incident/injury arises, a faculty will inform the director of operations about the emergency, who will then categorize the injury into three levels of injury severity: mild, moderate, and severe. For a mild injury such as a small cut or needle stick, the injured party will go to occupational health, a clinic for staff in the same building as the simulation center. For an injury of moderate severity, where the injured party can ambulate, the simulation team will escort the individual to the emergency room. For a severe injury where the injured party cannot ambulate, the simulation center will activate your local emergency system. In the case study provided, the fall and arm injury would be classified as moderate since the faculty member was able to walk and be taken to emergency care. Table 1, titled Simulation Safety Risks was developed through collaboration of the simulation team, educators and clinical staff with backgrounds in Emergency Medicine and Obstetrics and Gynecology. Each group brought its expertise to the Simulation Safety Risks table with clinical staff sharing insights on real world injuries, and simulation staff. This collaboration enabled us to develop a resource for managing physical safety while addressing both practical and educational aspects of safety in healthcare simulation settings.

**Table 1:** Simulation safety risks

Hazard	Categorize level	Plan of action
● Scratch ● Bruises	Mild Injury	1. Clean the wound
● Cuts ● Laceration ● Blood and Body ● Fluid Exposure	Moderate Injury	1. Clean the wound 2. Escort the injured party to the emergency room (only the injured party can ambulate)
● Fracture ● Chest Pain ● Shortness of breath ● Bruns	Severe Injury	1. We will call emergency services

Remember to document all injuries that happen and follow up with the injured party

A proactive approach to physical safety may be executed when developing curricula as part of normal processes and procedures. When considering the experiential portion of the class, consider the room size, the location of the equipment, and the number and position of the learners to help estimate how safe the simulation encounter will be. Dry running the scenario can help identify any miscalculations in meeting learning objectives and highlight any unexpected physical risks such as faulty equipment. Altering a simulation scenario drastically on the fly may create unanticipated psychological safety risks but physical ones as well. Familiarity with how the scenario is normally run and being mindful of its safety considerations will minimize most risk.

Along with a proactive approach should be a reflective strategy. The review of safety incidents, aligned with continuous improvement, within smaller programs, may be done by faculty and staff. As our program spans multiple locations, a centralized decision-making body has been formed to add in this review process. This Simulation Safety Advisory Council consists of multiple members, each responsible for representing their facility as the simulation safety leader. The facility lead conducts workplace inspections and safety audits as preventative strategies as well as documents any accident or near miss that occurred during simulation with the goal of co-developing strategies for corrective actions to prevent recurrence of similar incidents in the future [20].

### Simulation for safety systems evaluation

Using simulation for the purpose of identifying and mitigating risk in our scenarios is a process of risk aversion. Simulation may also be used to look at the process of physical safety breach response in order to prepare for the unexpected. The Donabedian Approach recommends the inclusion of training in its Process phase [13]. Similar to the re-creation of common and rare events and reflecting on the experience used by healthcare simulation, curricula applying simulated emergencies in the classroom can better prepare staff and faculty, as well as inform holes in the safety response policies. In other areas of safety, practice is common, and practice drills are recommended by the Occupational Safety and Health Administration to keep employees prepared [21]. For example, a fire drill is a simulation to practice the emergency evacuation procedure in the event of a fire. Since emergencies are rare in the classroom setting, staff need training to implement an adequate response. Like other curricula, performing needs assessments and collecting outcomes data around knowledge, skills, and attitudes can measure the impact of training sessions on physical safety awareness [22].

Using the policy as the backbone of the curriculum offers a guide for content. Focusing on the importance, the “why”, of safety will promote engagement. Addressing the different injury levels, the safety phrase, and reporting mechanism can help reinforce behaviors and skills needed to support the safety culture. Using simulation encounters as a means to explore safety, with managing physical emergencies (i.e., syncope, electrocution, falls, etc.) as the objective, can reinforce the strategies and better socialize the guidance offered by the policy. We recognize it is impossible to re-create all potential injuries; however, these experiences create a platform from which a conversation can build both awareness and explore varied approaches around physical safety [23]. Had we simulated some of the situations mentioned above, the case study presented may have been managed more efficiently and potentially avoided in its entirety.

### Physical safety system outcomes

Measurement of safety strategies and their effectiveness partnered with wide reporting promotes the inculcation of safety and is the product of organizational structure and process [24]. While latent safety threats during simulation are widely reported in the literature [25, 26], there is little guidance on physical safety reporting in healthcare simulation. Through reporting, there is the opportunity to identify physical safety threats that might be avoided at other institutions. Potential data may come in the form of workplace injury reporting or intentional safety scavenger hunts to identify threats. Trends in these reports, if injury is appropriately reported, may indicate safety process and strategies are effectively addressing the risks identified.

Regular communication of safety events and reports build awareness in the community and may occur in person, at meetings or through electronic reporting. Reports from a safety council should also be shared to aid in educator preparedness and preventative strategies. At our institution, a report was created to explore the use of the safety phrase, “Stop-Play” across the system. The investigation identified the phrase was most frequently used to address general safety concerns, sharps safety and fall risks. This feedback informs that process is being applied and mitigating risk effectively across multiple institutions. Moving the conversation beyond institutional reporting is to share with the larger community of practice, echoing the work of the Foundation for Healthcare Simulation Safety.

### Call to action for physical safety

The Donabedian Framework offers an organized approach to safety that considers structure, process, and outcomes to promote explicit conversations about physical safety within our simulation community. By creating a culture that promotes physical safety within healthcare simulation programs, we can prevent adverse events and keep education focused on education. As the practice of healthcare simulation grows with the increasing demand, we may find increased incidences of injuries related to the physical nature of the practice.

Physical simulation safety is a growing field and more research and reporting needs to be done in this area. Since these events are rare, simulation educators and societies should collaborate to develop shared safety approaches and standardized recommendations. Program improvement projects and publications focusing on physical safety can also drive awareness and motivation. Our conceptual framework offers guidance in creating recommendations around structural considerations, process and policy approaches and outcomes reporting to keep education physically safe and avoid future injuries to educators and learners.

## Conclusion

Through this article, we adapted the Donabedian Model to approach physical safety in the field of Healthcare Simulation. The model offers guidance around structural organization and processes that create systems to positively impact physical safety in learning environments. With this model, we hope to contribute to the growing body of work and conversations that are considering physical safety in experiential learning. Physical safety should not be something we worry about in created learning experiences and applying the Donabedian Model to physical safety in Healthcare Simulation, we can more confidently maintain focus on meeting our learning objectives.

## Data Availability

The datasets used and/or analyzed during the current study are available from the corresponding author on reasonable request.
